# Palliative Stereotactic Body Radiation Therapy for the Treatment of Refractory Ventricular Tachycardia

**DOI:** 10.7759/cureus.80001

**Published:** 2025-03-03

**Authors:** Kathleen Trinh, Aretha Kou

**Affiliations:** 1 Internal Medicine, University of California Los Angeles, Los Angeles, USA

**Keywords:** novel therapies, palliative radiation therapy, refractory ventricular tachycardia (vt), sbrt (stereotactic body radiotherapy), ventricular tachycardia (vt)

## Abstract

Stereotactic body radiation therapy (SBRT) is a promising noninvasive treatment for ventricular tachycardia (VT) that delivers targeted ablative energy to arrhythmogenic regions. Typically reserved for patients with VT refractory to conventional therapies, SBRT offers a viable option for individuals who are ineligible for invasive procedures due to high-risk profiles and multiple comorbidities. We report the case of a 62-year-old patient with refractory VT, deemed unsuitable for further interventions, who underwent SBRT and experienced a reduction in VT episodes. This case highlights the potential palliative application of SBRT in addressing the growing population of older patients with complex comorbid conditions.

## Introduction

Ventricular tachycardia (VT) ranges from benign, non-sustained episodes to sustained VT associated with hemodynamic collapse [1]. Patients with ischemic heart disease are at the greatest risk of sudden cardiac death from VT [2], making recurrent VT a significant clinical challenge with high morbidity and mortality. Mainline therapies for VT include antiarrhythmic drugs, catheter ablation, and cardiac rhythm management devices, such as an implantable cardioverter-defibrillator (ICD). Despite their widespread use, these conventional treatments have limitations, with antiarrhythmic drugs showing limited efficacy, recurrent ICD shocks impairing myocardial function, and catheter ablation posing procedural risks [3]. Overall, managing VT in patients with ischemic structural heart disease is particularly challenging, especially when it becomes refractory to conventional therapies or when these treatments are no longer viable options. Stereotactic body radiation therapy (SBRT) is a novel radiation therapy for VT that delivers targeted ablative energy to arrhythmogenic substrate, including areas that may be inaccessible to catheter ablation in certain cases [4]. As a non-invasive procedure, SBRT minimizes procedural risks and is typically reserved for patients with VT refractory to or unsuitable for catheter ablation or ICD placement. While SBRT has been explored as an alternative treatment for VT, we present a case where it was instead employed as the last available therapeutic avenue. As more people live longer with comorbidities that limit their eligibility for advanced interventions, SBRT may increasingly serve as a palliative option for managing VT. Current literature demonstrates its utility in providing immediate antiarrhythmic palliation for critically ill patients with refractory VT and electrical storms [5]. This case aims to raise awareness of SBRT as a viable palliative approach for recurrent and refractory VT in such patients with advanced disease and limited treatment options.

This article was previously presented as a poster at the 2024 American College of Physicians Southern California Regions 1, 2, & 3 Annual Scientific Meeting on October 5, 2024.

## Case presentation

A 62-year-old male presented to an outside hospital with a wide complex tachycardia after eight ICD shock therapies. He has a history of ischemic cardiomyopathy complicated by heart failure with reduced ejection fraction (EF; 35-40%, New York Heart Association (NYHA) Class III), BMI of 26, prior cardiac arrest, recurrent VT with right-sided cardiac resynchronization therapy-defibrillator (CRT-D) placement, now abandoned and replaced with a single chamber ICD, coronary heart disease with percutaneous coronary intervention of the left anterior descending artery, paroxysmal atrial fibrillation (A-fib), end-stage renal disease (ESRD) on hemodialysis, and cirrhosis. Although all eight shocks were successful, he continued to have incessant VT. He was cardioverted to A-fib with a slow ventricular response and stabilized with intravenous amiodarone and mexiletine. In the preceding year, the patient experienced at least six episodes of monomorphic VT, including three instances of VT storm that necessitated hospitalization. The patient was deemed ineligible for ablation or heart transplantation due to the high risk associated with his multiple comorbidities, including ESRD and cirrhosis. Given his recurrent and refractory VT, he was transferred to our institution for palliative SBRT.

**Figure 1:**
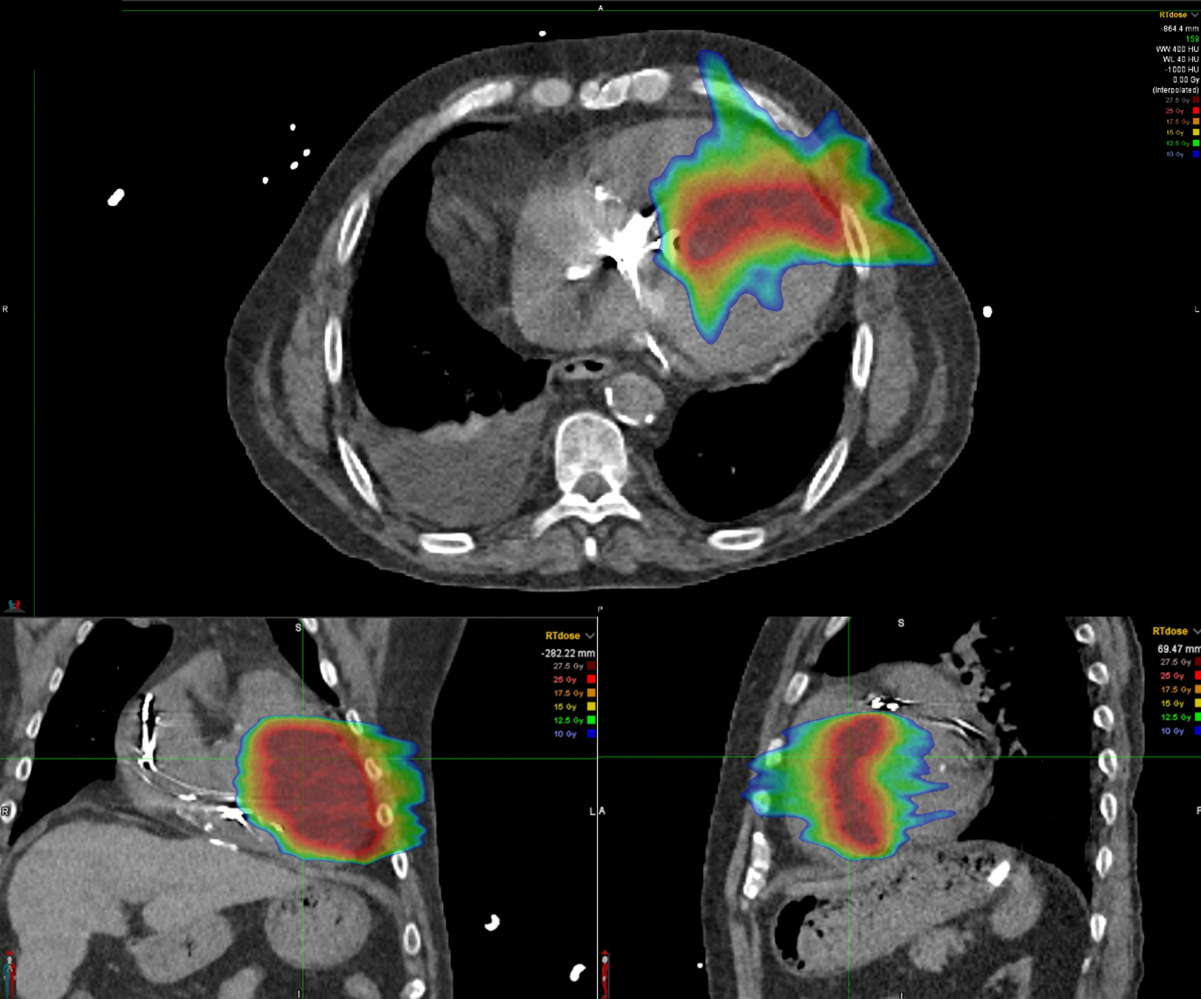
Stereotactic body radiation therapy treatment planning for ventricular tachycardia with computed tomography images, shown in axial (top), coronal (bottom left), and sagittal (bottom right) views. The color overlays represent the radiation dose distribution within the targeted myocardial region. The red zone corresponds to the highest dose (25 Gray), with decreasing doses represented by the orange, yellow, green, and blue areas (down to 10 Gray). The planning target volume is carefully delineated to ensure that the arrhythmogenic substrate is adequately covered by the therapeutic dose while minimizing exposure to adjacent structures at risk.

During his hospitalization, the patient’s regimen of amiodarone 400 mg BID and mexiletine 200 mg TID was continued, and his bradycardia gradually resolved. Cardiac magnetic resonance imaging (MRI) and a computed tomography (CT) simulation scan were performed to assist in radiation treatment planning (Figure 1). Due to his high-risk status, inpatient SBRT was scheduled for five days later. However, the initiation of SBRT was delayed by multiple complications, including a new left-sided groin hematoma and stenosis of his right arteriovenous fistula, which necessitated the placement of a right femoral tunneled hemodialysis catheter. He also experienced an episode of atrial flutter with rapid ventricular response, which converted back to A-fib after an amiodarone bolus and drip. Once he got stabilized, the patient underwent an uncomplicated single session of high-dose cardiac SBRT, receiving 25 Gy in a single fraction. He was discharged to his original institution in stable A-fib and restarted on low-intensity anticoagulation with heparin. At his three-month follow-up, he experienced one exacerbation of VT, which was attributed to hypokalemia.

## Discussion

We present a case demonstrating the use of SBRT as a palliative, last-resort treatment for refractory VT. Traditionally utilized in oncology, SBRT has emerged as a promising option for VT unresponsive to conventional therapy. While ICDs are effective in terminating VT, long-term recurrence rates remain above 50% [6]. Catheter ablation is recommended for patients with recurrent VT or intolerance to antiarrhythmic drugs. While effective, catheter ablation is an invasive procedure with limitations in accessing deeper myocardial sites, contributing to ablation failure [7]. Overall, VT recurs in 25%-50% of patients within the first year, necessitating additional procedures and ongoing use of antiarrhythmic drugs [1].

As treatment options become exhausted, cardiac SBRT can be considered. SBRT integrates multimodal mapping techniques to noninvasively deliver precise radiation to arrhythmogenic substrates, targeting areas too extensive or inaccessible for catheter ablation with maximum therapeutic efficacy while minimizing damage to surrounding healthy cardiac tissue [6]. Although late radiation toxicity and myocardial fibrosis are concerns with conventional radiotherapy, SBRT’s focused targeting, improved dose fall-off, and cardiac structure sparing may reduce both acute and long-term toxicities [8]. For treatment planning, nuclear imaging, MRI, and CT are commonly used; however, nuclear imaging’s poor spatial resolution has largely led to its supersession by cardiac MRI and CT [6]. Studies generally report substantial short-term reductions in VT burden (six weeks to six months after SBRT), despite differences in patient selection, imaging modalities for planning, and long-term monitoring [9]. Given its effectiveness in immediate arrhythmia control, SBRT serves as a valuable option for providing rapid antiarrhythmic palliation in cases like ours [5]. Although long-term outcomes raise concerns about recurrence and mortality, with published literature reporting a high long-term recurrence rate and a cumulative VT-free survival of 38.6% [9], emerging data suggest the potential for sustained efficacy and tolerance in reducing VT episodes. Studies on one-year outcomes following SBRT have demonstrated a reduction in treated VT episodes [10,11]. Additionally, ongoing clinical studies, including the RAVENTA trial and STRA-MI-VT study, are actively assessing the risks and benefits of SBRT, with preliminary data indicating its potential efficacy in VT management [12,13].

**Figure 2:**
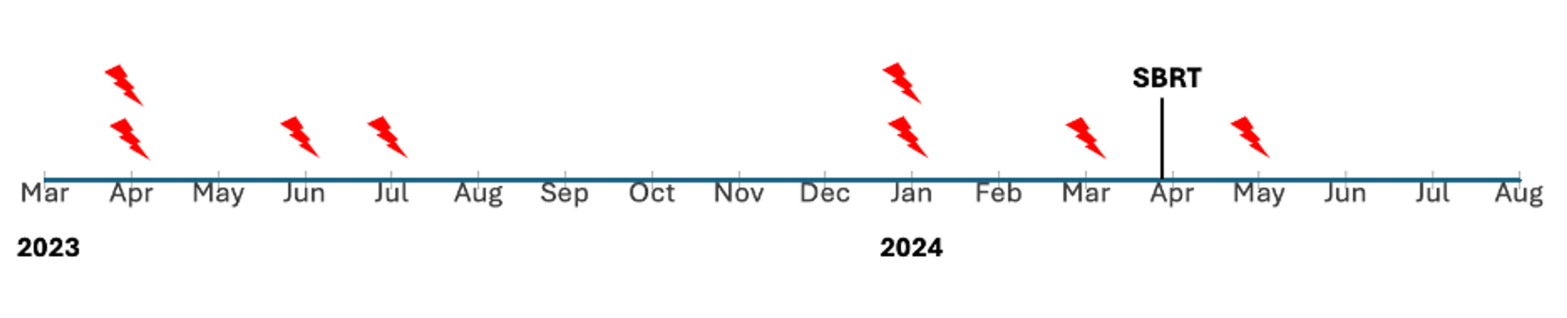
Timeline of ventricular tachycardia recurrences before and after stereotactic body radiation therapy.

Our patient represents a severe case, characterized by multiple comorbidities, previous device placements, and ineligibility for catheter ablation or advanced therapies, such as heart transplantation. SBRT was ultimately pursued as a palliative measure to reduce VT severity and improve quality of life. He experienced one VT recurrence in the three months after SBRT, a decrease from three in the preceding three months (Figure 2), in line with his palliative goals. This aligns with outcomes seen in critically ill patients with high disease burdens, who experience a reduction in the overall number of VT episodes despite a notable recurrence rate [14]. This case highlights the role of SBRT in palliative care. Palliative success is achieved by reducing episode burden to alleviate symptoms associated with non-curable medical conditions. As life expectancy increases, many patients face comorbidities that render them ineligible for invasive or curative treatments. SBRT may be integrated into patient selection algorithms to guide treatment decisions based on therapeutic goals (palliative vs. curative) and patient candidacy, offering a valuable option for managing symptomatic VT.

## Conclusions

VT is a life-threatening condition, particularly when advanced therapeutic options are no longer feasible. SBRT has emerged as an innovative alternative treatment for managing VT when conventional therapies, such as antiarrhythmic drugs or ICD shocks, are ineffective. While the novelty of SBRT and the absence of standardized protocols highlight the need for further investigation, current evidence supports its promise as a viable therapeutic option. It offers the added benefits of minimizing procedural risks compared to catheter ablation and reaching otherwise inaccessible arrhythmogenic sites. SBRT has been shown to significantly reduce VT episodes, especially in the early post-procedure period. As the aging population with increasing comorbidities grows, SBRT is poised to play a crucial role as a palliative treatment option for patients with limited alternatives. This case illustrates the potential of SBRT to provide rapid antiarrhythmic palliation in critically ill patients facing untreatable and refractory VT. Greater awareness of the patient demographic eligible for SBRT may help minimize delays in treatment and contribute to improved outcomes for this high-risk population.
